# Antimicrobial therapy in neonatal intensive care unit

**DOI:** 10.1186/s13052-015-0117-7

**Published:** 2015-04-01

**Authors:** Chryssoula Tzialla, Alessandro Borghesi, Gregorio Serra, Mauro Stronati, Giovanni Corsello

**Affiliations:** Neonatologia, Patologia Neonatale e Terapia Intensiva neonatale, Fondazione IRCCS Policlinico San Matteo, Piazzale Golgi 19, 27100 Pavia, Italy; Terapia Intensiva Neonatale Dipartimento di Scienze per la Promozione della Salute e Materno Infantile “G. D’Alessandro”, Università degli Studi di Palermo, Via Alfonso Giordano, 390127 Palermo, Italy

**Keywords:** Newborn, Antibiotic, Neonatal sepsis, Resistant bacteria, Empiric therapy, Antibiotic stewardship

## Abstract

Severe infections represent the main cause of neonatal mortality accounting for more than one million neonatal deaths worldwide every year. Antibiotics are the most commonly prescribed medications in neonatal intensive care units (NICUs) and in industrialized countries about 1% of neonates are exposed to antibiotic therapy. Sepsis has often nonspecific signs and symptoms and empiric antimicrobial therapy is promptly initiated in high risk of sepsis or symptomatic infants. However continued use of empiric broad-spectrum antibiotic treatment in the setting of negative cultures especially in preterm infants may not be harmless.

The benefits of antibiotic therapy when indicated are clearly enormous, but the continued use of antibiotics without any microbiological justification is dangerous and only leads to adverse events. The purpose of this review is to highlight the inappropriate use of antibiotics in the NICUs, to exam the impact of antibiotic treatment in preterm infants with negative cultures and to summarize existing knowledge regarding the appropriate choice of antimicrobial agents and optimal duration of therapy in neonates with suspected or culture-proven sepsis in order to prevent serious consequences.

## Introduction

Sepsis represent the main cause of neonatal mortality accounting for more than one million neonatal deaths worldwide every year, and antibiotics are the most commonly prescribed medications in the neonatal intensive care units (NICU) [1,2]. Sepsis has often nonspecific signs and implies in serious consequences; as a result, empirical antimicrobial therapy is promptly initiated in symptomatic infants with suspected sepsis after obtaining biological material for culture [1]. However, neonates who do not have infection often receive antimicrobial agents during hospital stay, and inappropriate empirical antibiotic treatment may have serious side effects [3].

Nearly all extremely low birth weight infants (ELBW) infants admitted to a NICU receive an empirical antibiotic treatment in the first postnatal days, in spite of sterile cultures and low incidence of culture-proven bacterial sepsis in this population [1,3,4]. This observation has been confirmed by a study of the *National Institute of Child Health and Human Development National Research Network* on 6956 very low birth weight (VLBW) infants, showing that 56% of all infants received at least one course of antibiotic treatment, even if proven sepsis was diagnosed in only 21% of all infants [5].

In this review we describe the use of antibiotics in the NICU, focusing on the potential serious adverse effects of inappropriate use; we identify the opportunities for improving antibiotic prescription in the NICU, and we discuss the future directions of antimicrobial therapy.

### Epidemiology of bacterial infections in the NICU

Neonatal sepsis can be classified as early-onset (EOS) and late-onset (LOS) sepsis. EOS is most often caused by group B streptococcus (GBS) (43%), followed by *Escherichia coli* (15.5-29%). Among VLBW infants, the rate of *Escherichia coli* infection exceeds that of GBS infection (5.1 vs 2.1 per 1000 live births) [3].

LOS is mainly caused by Gram-positive bacteria (GPB) (49%), most often coagulase-negative Staphylococcus (CoNS) (45%). Gram-negative LOS is less common (23%), but is associated with greater mortality in the NICU (19-36%) [6,7].

As showed by various studies, antibiotics such as ampicillin, gentamicin and cefotaxime commonly used for empirical therapy appear to be appropriate. In a recent study, investigators revealed that more than 94% of the EOS isolates were susceptible to penicillin and gentamicin, to amoxicillin and cefotaxime and to cefotaxime alone. The LOS isolates (excluding CONS) had a more than 96% susceptibility to flucloxacillin or amoxicillin and gentamicin, to amoxicillin and cefotaxime, but only 78% to cefotaxime alone. The investigators concluded that cefotaxime should not be included in the empiric regimen of suspected sepsis, because of lower susceptibility levels [7].

Blackburn *et al.*, in a study of neonatal septicemia found that only 1.4% of Gram-negative bacteria (GNB) were resistant to penicillin plus gentamicin, whereas 10.4% of isolates tested against amoxicillin plus cefotaxime were resistant to this association [8].

Most hospital acquired CoNS are resistant to many commonly prescribed antibiotics. In the NICU, enterococci are less frequently isolated than staphylococcal species. Nevertheless, ampicillin-resistant, and, more recently, vancomycin-resistant enterococci have been described and have become endemic in some NICUs [9].

GNB are often resistant to at least one class of antibiotics usually used, and bacteria that are multi- or extensively-resistant to conventional antibiotics are frequently isolated. Pan-resistant pathogens are rarely isolated in the NICUs, where resistance is most frequently found to piperacillin-tazobactam, ceftazidime, and/or gentamicin [9,10].

Moreover, the emergence of extended-spectrum β-lactamase (ESBL)-producing GNB confers resistance to penicillins and cephalosporins, often coexisting with resistance to other antibiotic categories as fluoroquinolones and aminoglycosides [9,11].

### Risks associated with empirical administration of broad-spectrum antibiotics

The use of broad-spectrum antibiotics is associated with different adverse effects: alteration of gut colonization, emergency of resistant strains, and increasing risk of Candida colonization and subsequent invasive candidiasis [4].

All antibiotics can alter gut colonization of the patient, promoting either antibiotic resistance among normal commensal organisms or the emergence of other pathogens [4,12].

A number of *in vitro* and *in vivo* studies have shown that, although short courses of carbapanems and third-generation cephalosporins cover a broad spectrum of bacteria, their prolonged and intensive use selects resistant bacteria. Overuse of third-generation cephalosporins favors the emergence of ESBL-producing strains of GNB in NICUs [10,13].

In order to study the effects of empirical antibiotics on the emergence of resistant pathogens, de Man *et al.* [14] examined 436 infants admitted to 2 NICUs who were assigned initially to either a narrow-spectrum antibiotic regimen (penicillin or flucloxacillin plus tobramycin) or a broad-spectrum regimen (amoxicillin plus cefotaxime) and exchanged regimens after 6 months. The investigators demonstrated that the relative risk for colonization with strains resistant to empirical therapy per 1000 patients at risk was 18-fold higher in the broad-spectrum regimen group than in the narrow-spectrum regimen group.

Exposure to broad spectrum antibiotics has been also associated with the emergence of invasive candidiasis. In a cohort of 3,702 ELBW infants, previous use of third generation cephalosporins or carbapenems were associated with an increased risk of invasive candidiasis (OR 2.2, 95% CI 1.4-3.3). The incidence of candidiasis between centers varied from 2.4% to 20.2% and correlated with the average number of days of broad spectrum antibiotic use per infant with sterile cultures throughout hospitalization [15].

A multicenter cohort study of 128,914 neonates, revealed that the use of ampicillin/cefotaxime during the first 3 days after birth is associated with an increased risk of death before discharge (OR 1.5, 95% CI 1.4-1.7) compared with the use of ampicillin/gentamicin, even if the authors highlighted that this observation may be limited by selection bias. The authors concluded that, for patients receiving ampicillin, the concurrent use of cefotaxime during the first three days after birth is either a surrogate for an unrecognized factor or is itself associated with an increased risk of death, compared with the concurrent use of gentamicin [1].

### Adverse effects of prolonged courses of empirical antibiotic treatment

For culture-proven sepsis a full course of antibiotics is indicated. Conversely, concerns remain about the optimal length of antibiotic therapy for clinical, not microbiologically demonstrated sepsis. Recent cohort studies show an association between the duration of empirical antibiotic therapy and mortality, necrotizing enterocolitis (NEC) and LOS.

Cotten *et al*. [16] conducted a retrospective cohort analysis of 5,693 ELBW infants admitted to 19 tertiary centers. Of 5,693 infants, 4,039 survived >5 days, received initial empirical antibiotic treatment and had sterile initial blood culture at 72 hours of life. In a multivariate analysis adjusted for risk factors, prolonged duration of therapy was associated with increased odds of NEC or death or death alone. Each additional day of antibiotic therapy was associated with a 4% increase in the odds of NEC or death, a 7% increase in the odds of NEC alone and a 16% increase in the odds of death alone.

A retrospective 2:1 control case analysis examined the association between antibiotic exposure and the risk of NEC. When neonates with sepsis were removed from cohort, antibiotic duration increased the risk of NEC by approximately 20% per day of exposure (OR = 1.2). Exposure for >10 days resulted in nearly a threefold increase in the risk of developing NEC [17].

Prolonged antibiotic therapy has also been associated with LOS. A retrospective study of 365 infants ≤32 weeks gestational age (GA) and ≤1500 g birth weight (BW), who survived free of sepsis and NEC in the first week of life, found that prolonged antibiotic therapy (≥5 days) initiated on the day of birth was independently associated with LOS alone and the composite outcome of LOS, NEC or death. Each additional day of antibiotics was associated with a significantly increased risk of these outcomes. For infants who received any initial empirical antibiotic exposure, the adjusted attributable risk for LOS, NEC or death was 32% and the number needed to harm was 3 [18].

### Recommendations for a judicious use of antibiotics

#### Choice of the antibiotic agent

Concerning the newborn infants, there are no randomized controlled trials that can definitely prove the best choice of antibiotics. However, many authors agree that an association of a penicillin or semisynthetic penicillin (ampicillin) together with an aminoglycoside is effective against microorganisms causing EOS, and therefore can be considered the best empirical regimen [10,19,20].

For the treatment of suspected LOS, different authors agree that the best regimen is an antistaphylococcal penicillin (oxacillin, flucloxacillin) together with an aminoglycoside; the choice of vancomycin should be restricted to microbiologically demonstrated cases of methicillin-resistant *Staphylococcus aureus* (MRSA) or CoNS [19,20].

In a recent review, Sivanandan *et al*. [21] recommended the same antibiotic combinations for empirical therapy of EOS and LOS in neonates. In case of LOS in an instable neonate and in areas where MRSA is prevalent, vancomycin and a third-generation cephalosporin should be considered. For the treatment of suspected early-onset meningitis the authors recommended a combination of ampicillin and aminoglycoside or ampicillin and cefotaxime, and in case of late-onset meningitis a combination of an antistaphilococcal antibiotic (nafcillin or vancomycin) plus a third-generation cephalosporin with or without aminoglycoside.

Russel et al. [12] for the therapy of meningitis suggest as first line treatment the combination of cefotaxime and amoxicillin with or without gentamicin.

Different authors state that empirical therapy should never be started with a broad spectrum antibiotic such as a third-generation cephalosporin or a carbapenem, and their use should be restricted to particular cases [13,19]. Gray *et al.* [10] suggest the use of piperacillin-tazobactam in the units where aminoglycoside-resistant GBN are prevalent as an alternative to third generation cephalosporins.

Russel *et al.* in a recent review, based on epidemiological data from UK neonatal infection surveillance studies, suggest for EOS and LOS treatment, antibiotic strategies reported in Table 1 [12].

**Table 1:** **Choice of antibiotics**

**EOS**	Penicillin + gentamicin
	- if *Listeria monocytogenes*: amoxicillin + gentamicin
	- if *S.aureus*: flucloxacillin + gentamicin
**LOS**	*First line*: flucloxacillin + gentamicin
	*Second line:*
	- vancomycin + gentamicin (with caution)
	- vancomycin + piperacillin/tazobactam (to extend Gram-negative cover)
	*Third line*: meropenem, ciprofloxacin
**Meningitis**	*First line*: cefotaxime with amoxicillin ± gentamicin
	*Second line*: meropenem
**Gram positive multiresistant bacteria**	Currently: glycopeptide antibiotics are the mainstay of therapy, especially vancomycin; if necessary linezolid, clindamycin, rifampicin and daptomycin could be alternative regimens
	In the future: novel cephalosporins like ceftaroline and ceftobiprole; novel lipoglycopeptide antibiotics are oritavacin and dalbavancin; telavacin has been approved in the USA in adults
**Gram negative multiresistant bacteria**	Currently: aminoglycosides and cephalosporins are the antibiotics of choice; carbapenems, colistin, co-trimoxazole, ticarcillin-clavulanic acid could be the an alternative; fluoroquinolone, ciprofloxacin, tigecycline and tetraciclins could only be justified in extreme cases.
	In the future: treatment options are extremely limited

Modified from: Russell AB, Sharland M, Heath PT. Improving antibiotic prescribing in neonatal units: time to act. *Arch Dis Fetal Neonatal* 2012; 97:F141-146 and Gray JW, Patel M. Management of antibiotic-resistant infection in the newborn. *Arch Dis Child Educ Pract* 2011 Aug;96(4):122–7.

#### Duration of the antibiotic course

Culture-proven neonatal sepsis is treated with full course of appropriate antibiotics; the appropriate duration of the antibiotic course is more difficult to be established in case of suspected (clinical) sepsis with negative cultures. Usually, antibiotics are discontinued as soon as blood cultures are confirmed negative (48–72 hours), and if laboratory results and the evolution of the clinical signs allow to exclude an infection [15,21-24].

In case of abnormal laboratory tests [white blood cell count and C-reactive protein (CRP) at age 6–12 h] in well-appearing neonate with negative blood culture Polin and the Committee on Fetus and Newborn [11] (COFN) suggest to continue empiric antibiotic therapy if mother received antibiotics during labor and delivery in case of infants <37 weeks’ gestation with risk factors for sepsis and infants ≥ 37 weeks’ gestation born from mothers with chorioamnionitis.

The algorithms for duration of empiric therapy when cultures are sterile suggested by COFN generated discussion about the lack of strong supportive evidence to guide decisions to stop antimicrobials at 48 h in certain cases.

Cotten *et al*. [25] in a recent review highlight that current studies are inadequate to specify appropriate testing a timing of diagnostic tests in all situations in which empirical therapy have been started. The authors offer the following suggestions for the management of term and late preterm neonates on empirical therapy for EOS with negative cultures at 48 postnatal hours: i) continuation of treatment for 7 days if clinical signs of sepsis persist over 24 hours; ii) stop antibiotics at 48 hours in asymptomatic neonates with initial (4 postnatal hours) normal complete blood count (initial laboratory tests drawn by risk factors), and in neonates with transient clinical signs (lasting less than 24 h), and abnormal initial complete blood count, if serial CRP measurements at 24 and 48 hours are low in a well-appearing neonate.

In case of culture-proven sepsis, Sivanandan *et al*. [21] suggest that it is reasonable to treat for 10–14 days with appropriate antimicrobial agents infants with blood culture-proven sepsis without meningitis. However, in selected situations [i.e. neonates >32 weeks GA and >1500 g BW, who became asymptomatic with 5 days of appropriate therapy], it is reasonable to discontinue antibiotics at 7–10 days if laboratory results are normal and cultures are sterile in a well-appearing child. For neonatal meningitis, the same authors suggest a duration of therapy of 14 to 21 days for GBS, ≥ 21 days for L*ysteria monocytogenes*, a minimum of 21 days for Gram-negative meningitis and 4 to 6 weeks in cases complicated with intracranial abscesses.

### Therapies for resistant pathogens

Even though an increase in vancomycin MIC values, within the susceptible range, has been registered among isolates of MRSA, CoNS or *S. aureus* strains vancomycin-intermediate or vancomycin-resistant have not been isolated from a NICU population so far. Consequently, glycopeptides remain an appropriate treatment for most staphylococcal infections in this setting [9,26]. However in case of unresponsive Gram-positive infections, linezolid has been the most used in neonatology, even if the use of daptomycin have been described in few reports in case of persistent staphylococcal bacteremia in neonates [26].

Several novel antibiotics active against GPB are currently in diverse phases of development and clinical trials are ongoing. In particular, advanced-generation cephalosporins like ceftaroline and ceftobiprole with activity against multidrug-resistant staphylococci have been reported in adults [26,27], as well as lipoglycopeptides agents with activity against multidrug-resistant gram-positive pathogens like oritavancin and dalbavancin and telavancin. All three agents are promising alternatives for the treatment of complicated skin and soft-tissue infections in adults but there are no data about their pharmacokinetics in neonates [26,28].

For serious antibiotic-resistant GNB infections, carbapenems have become the mainstay of treatment with meropenem being the most widely used and doripenem as a newer carbapenem with greater activity against *Pseudomonas aeruginosa* [10]*.* However rapid emergence of resistance to these antibiotics means that the use of agents such as colistin, fosfomycin and tigecycline must be considered. Colistin is largely experienced in the neonatal population, but it must be kept in mind that is not effective against *Proteus* and *Serratia*. There is little experience of using fosfomycin in neonates but is worth considering as a final-resort therapy for extensively drug-resistant GNB [10,26]. Tigecycline, active against hard to treat pathogens like many multidrug-resistant GPB e GNB, is inactive against *Pseudomonas Aeruginosa* [10,29], but due to the possible effects on the bone growth in children, the use in neonates could only be justified in extreme cases (Table 1) [10,26].

### Future strategies

#### Appropriate antibiotic policies

Antimicrobial stewardship programs (ASP) were introduced in the 1980s, with the aim to reduce unnecessary therapies. Nevertheless, only in 2007, the Infectious Diseases Society of America, together with other professional organizations, published guidelines in order to implement multidisciplinary ASP [30]. However, in spite of positive experiences on adults, data about the consequences of ASPs in neonatal settings are lacking.

Recently, several authors suggested different strategies that might be helpful in a NICU that include implementation of systems for surveillance of bloodstream infections, education of practitioners concerning the development of resistance, use of narrow spectrum empirical antibiotic policy and stop of empirical treatment or documented justification for continuation when blood cultures are negative, use of narrowest spectrum antibiotics for a proven infection, formulary restriction and pre-authorization requirements for selected antimicrobial agents like cephalosporins, meropenem, vancomycin and teicoplanin [11,12].

Patel *et al.* [31] suggest that although specific guidelines for neonates are often lacking, antibiotic stewardship principles like those proposed by the Get Smart for Health Care Campaign of the Centers for Disease Control and Prevention can be applied to the NICU along with the development of an interdisciplinary antimicrobial stewardship team and metrics to measure successful implementation of ASP.

#### Development of innovative treatments

Spellberg *et al.* [32].suggest future strategies to combat antibiotic-resistance like therapies with diminished potential to drive resistance (pe infusion of monoclonal antibodies and white cells that kill microbes or biologic agents that alter bacterial ability to trigger inflammation) and treatments that alter host-microbe interactions like moderation of host inflammation and limitation of microbial growth (pe sequestration of host nutrients, probiotics administration that compete with microbial growth).

#### Discovery of new antibiotics

The discovery of new antibiotics must face a number of challenges that make the development of new antibiotic drugs more difficult compared to other non-antibiotic drugs. These have been well summarized in a recent review by Lewis *et al.* [33].

First, the poor penetration of antibiotics in prokaryotic cells requires the delivery of higher amounts of a compound which, in turn, increases the risk of toxicity and narrows the therapeutic range.

In addition, specifically targeting GNB is even more challenging, as not only the inner membrane restricts the penetration of hydrophilic substances, but also the outer membrane further reduces the number of compounds that may be effective, and the multidrug-resistant pumps extrude any compounds that leak in through the outer membrane.

Even when these pharmacodynamics-related issues are resolved, the development of a new drug needs to face the pharmacokinetics-related issues; indeed, the search for molecules with physicochemical properties to improve the likelihood of bioavailability (e.g. by applying the Lipinski’s rules) may not match with the need of a compound with physicochemical properties that improve penetration into prokaryotes.

Once discovered, the compound must be tested in clinical trials; however, the identification and recruitment of patients infected with multi-resistant bacteria may be difficult, as most infections are caused by pathogens susceptible to the available compounds.

Finally, there is modest return on investment on antibiotic development compared to other drugs. Indeed, antibiotic therapy is typically short-term, lasting only some days, while therapies with cholesterol-lowering drugs or with anti-hypertensive drugs last for years, or lifelong; and, in any case, resistance to the new antibiotics will eventually develop, limiting their use and the profits that it produces.

Despite all these considerations, most of the potential bacterial targets for antibiotics are still unexploited. It is assumed that there are approximately 200 conserved essential proteins in bacteria, but the current antibiotics only hit few targets or pathways [33]. Future efforts should focus on the discovery of compounds directed against these new targets.

## Conclusions

Sepsis represents the main cause of neonatal mortality and antibiotics are the most commonly prescribed medications in the NICUs. Wise choice of antimicrobial agents and optimal duration of therapy in neonates with suspected or culture-proven sepsis is crucial in order to limit the use of unnecessary broad spectrum antibiotic therapy, and to provide local solutions to the world-wide race against antimicrobial resistance. While there is an increasing choice of drugs for treating multiresistant GPB, alternatives for GNB will be seriously restricted for the foreseeable future. This makes important the need to promote attitudes that fortify measures for infection-control and to develop new treatments that complement traditional approaches.

## Abbreviations

NICU: Neonatal intensive care units
ELBW: Extremely low birth weight infants
VLBW: Very low birth weight
EOS: Early-onset sepsis
LOS: Late-onset sepsis
GBS: Group B streptococcus
CoNS: Coagulase-negative Staphylococcus
GPB: Gram-positive bacteria
GNB: Gram-negative bacteria
ESBL: Extended-spectrum β-lactamase
NEC: Necrotizing enterocolitis
GA: Gestational age
BW: Birth weight
MRSA: Methicillin-resistant *Staphylococcus aureus*
CRP: C-reactive protein
COFN: Committee on Fetus and Newborn
ASP: Antimicrobial stewardship programs
